# The value of cholangioscopy-guided bite-on-bite (-on bite) biopsies in indeterminate biliary duct strictures

**DOI:** 10.1055/a-2619-6803

**Published:** 2025-06-25

**Authors:** David M. de Jong, Pieter Jan F. de Jonge, Pauline M. C. Stassen, Petko Karagyozov, Juan J. Vila, Ignacio Fernández-Urién, Martin W. James, Suresh V. Venkatachalapathy, Kofi W. Oppong, Andrea Anderloni, Alessandro Repici, Roberto Gabbiadini, Deepak Joshi, Mark Ellrichmann, Leena Kylänpää, Marianne Udd, Frans van der Heide, Pieter Hindryckx, Gareth Corbett, Kirill Basiliya, Vincenzo Cennamo, Stefano Landi, Simon Phillpotts, George J. Webster, Marco J. Bruno, on behalf of the European Cholangioscopy Group

**Affiliations:** 1 Department of Gastroenterology and Hepatology, Erasmus MC University Medical Center, Rotterdam, The Netherlands; 2 Clinic of Gastroenterology, Acibadem City Clinic University Hospital Tokuda, Sofia, Bulgaria; 3 Endoscopy Unit, Gastroenterology Department, Hospital Universitario de Navarra, IdiSNA, Navarra Institute for Health Research, Pamplona, Spain; 4 NIHR Nottingham Biomedical Research Centre, Nottingham University Hospitals NHS Trust, and School of Medicine, University of Nottingham, Nottingham, United Kingdom; 5 Department of Gastroenterology and Hepatology, Newcastle Upon Tyne Hospitals NHS Foundation Trust, Newcastle Upon Tyne, United Kingdom; 6 Gastroenterology and Digestive Endoscopy Unit, Fondazione IRCCS Policlinico San Matteo, Pavia, Italy; 7 Endoscopy Unit, IRCCS Humanitas Research Hospital, Milan, Italy; 8 Department of Biomedical Sciences, Humanitas University, Milan, Italy; 9 Institute of Liver Studies, King’s College Hospital NHS Foundation Trust, London, United Kingdom; 10 Interdisciplinary Endoscopy, Medical Department 1, University Hospital Schleswig-Holstein, Campus Kiel, Kiel, Germany; 11 Department of Gastrointestinal Surgery, Helsinki University Central Hospital, Helsinki, Finland; 12 Department of Gastroenterology and Hepatology, University of Groningen, University Medical Center Groningen, Groningen, The Netherlands; 13 Department of Gastroenterology and Hepatology, University Hospital of Ghent, Ghent, Belgium; 14 Department of Gastroenterology and Hepatology, Cambridge University Hospitals NHS Foundation Trust, Cambridge, United Kingdom; 15 Gastroenterology and Interventional Endoscopy Unit, Local Health Authority of Bologna, Bologna, Italy; 16 Department of Gastroenterology, University College London Hospitals NHS Foundation Trust, London, United Kingdom

## Abstract

**Background** Digital single-operator cholangioscopy (dSOC) has improved the diagnostic accuracy of indeterminate biliary duct strictures (IBDS) through targeted intraductal biopsy sampling. However, the optimal biopsy technique remains uncertain.

**Methods** This international, multicenter, prospective interventional study (November 2020–August 2022) included patients with IBDS undergoing dSOC. Stricture sampling involved obtaining at least four single biopsies and at least one bite-on-bite biopsy (BBB) in all patients. Definitive diagnosis was established by pathology outcomes and 1-year clinical follow-up. The primary outcome was the accuracy of both biopsy techniques.

**Results** 89 patients were included, with 76 hilar strictures and 13 distal strictures. Technical success for obtaining adequate tissue samples was 82/89 (92.1 %) for single biopsies and 78/89 (87.6 %) for BBB. Malignancy was confirmed in 31/82 (37.8 %) and 29/78 (37.2 %) cases in single biopsies and BBB, respectively. Among 76 patients in whom both techniques were successful, pathology results were discordant in three cases (3.9 %), primarily due to understaging by BBB. Among 82 patients with complete follow-up, malignancy was confirmed in 51 (62.2 %). Sensitivity, specificity, and accuracy for malignancy or high grade dysplasia were 66.0 %, 100 %, and 78.8 % for single biopsies, and 63.8 %, 100 %, and 77.6 % for BBB, respectively. Sensitivity and accuracy were significantly decreased after stent placement or intraductal tissue acquisition during prior ERCP. The number of BBBs did not impact sensitivity or accuracy.

**Conclusions** BBB did not outperform at least four single biopsies for IBDS. Prior manipulation of IBDS, through stent placement or prior tissue acquisition, was associated with a decreased diagnostic yield.

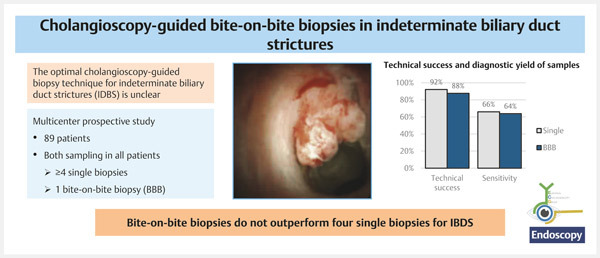

## Introduction

Indeterminate biliary duct stricture (IBDS) frequently poses a diagnostic challenge, and an accurate diagnosis remains problematic 1 . Traditional diagnostic methods such as endoscopic retrograde cholangiopancreatography (ERCP) with brushing or fluoroscopy-guided intraductal biopsies have a high specificity, but low sensitivity, for the diagnosis of malignancy 2 . A number of benign conditions, such as primary sclerosing cholangitis and IgG4-related sclerosing cholangitis, frequently mimic malignancy and differentiation can be challenging 3 . Approximately 15 % to 25 % of patients who undergo surgical resection for biliary strictures because of presumed malignancy, without prior pathological diagnosis, have benign disease at post-surgical pathological examination 4 5 . Hence, improvement in defining the etiology of a biliary stricture is critical for improving appropriate curative surgical resection rates for cancer and reducing the number of unnecessary surgical resections or explorative surgeries.

Digital single-operator cholangioscopy (dSOC) has improved the detection rate of malignancy through direct stricture visualization and the ability to obtain targeted biopsies 6 7 . However, the diagnostic accuracy of cholangioscopic visual diagnosis has been shown to be suboptimal, and therefore obtaining a tissue diagnosis remains mandatory 8 . There is limited evidence and currently no consensus on the optimal biopsy strategy when performing dSOC in patients with IBDS 9 , with data suggesting that obtaining at least three single biopsies yields more adequate specimen samples for pathological examination 10 . However, improved forceps designs, such as the SpyBite Max forceps (biopsy jaw outer diameter 1 mm; Boston Scientific, Marlborough, Massachusetts, USA), can obtain larger tissue samples, but improved diagnostic accuracy has not yet been shown 11 12 . Studies on bite-on-bite biopsies (BBB) for (residual) esophageal cancer 13 and subepithelial lesions 14 15 have suggested a superior yield for establishing a definitive pathology diagnosis compared with single biopsies, albeit with a large range. It has been suggested that instead of obtaining single biopsies, BBB may obtain larger and deeper bile duct samples and have a higher diagnostic yield for diagnosis of malignancy in patients with IBDS.

To date, no studies have systematically compared the diagnostic yield of four single biopsies with that of BBB in patients with IBDS. We aimed to assess the feasibility and diagnostic yield of tissue sampling via multiple dSOC-guided biopsies and to analyze the added yield of the bite-on-bite (-on-bite) technique in a representative study population with a high pretest probability of malignancy.

## Methods

### Study design and population

An international, multicenter, prospective interventional study was performed in patients who underwent dSOC for the evaluation of IBDS between November 2020 and August 2022. All 14 participating centers were members of the European Cholangioscopy Group, including therapeutic endoscopists with significant experience of dSOC (> 200 lifetime procedures). All centers obtained ethical approval from their respective local ethics committees. The study was conducted according to the principles of the Helsinki Declaration, and followed the Strengthening the Reporting of Observational studies in Epidemiology (STROBE) reporting guidelines (see **Table 1 s** in the online-only Supplementary material).

Eligible patients were diagnosed with an IBDS, defined as a bile duct stricture or filling defect of indeterminate nature after previous laboratory work-up, abdominal imaging, ERCP or endoscopic ultrasound (EUS), and/or negative or inconclusive cytology or histology after ERCP with brush cytology or fluoroscopy-guided intraductal biopsies, and/or despite indefinite cytology or histology findings with persistent suspicion of a malignant etiology based on clinical symptoms such as unexpected weight loss, loss of appetite, and obstructive jaundice. Other inclusion criteria were 18 years of age or older, and willingness and ability to provide written informed consent for participation. Patients were not eligible when a contraindication to ERCP, dSOC, or biopsy was present.

### dSOC procedure



**Video 1** Cholangioscopy-guided single biopsy and bite-on-bite biopsies in indeterminate biliary duct strictures.


**Fig. 1:**
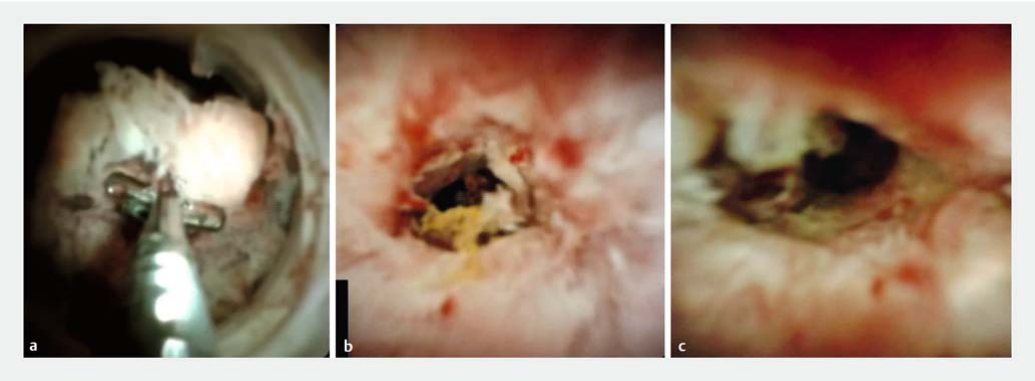
Three examples of indeterminate biliary duct strictures with digital single-operator cholangioscopy. **a** A perihilar stricture. Multiple single biopsies showed benign disease, while bite-on-bite biopsy (BBB) yielded inadequate tissue. Finally, endoscopic ultrasound-guided biopsy confirmed the suspicion of perihilar cholangiocarcinoma. **b** A distal stricture located at the insertion of the cystic duct. Both single and BBB biopsies were inconclusive. Surgical resection confirmed chronic segmental sclerosing cholangitis. **c** A perihilar stricture. Both single and BBB biopsies were suspicious for malignancy.

Procedures were performed with the patient under conscious sedation, propofol sedation, or general anesthesia. Cholangioscopy was performed with a digital single-operator cholangiopancreatoscopy system (SpyGlass DS II Direct Visualization System; Boston Scientific), which was advanced either over a 0.025– or 0.035-inch guidewire, or freehand into the common bile duct (CBD) up to the level of the target stricture. Examples of IBDS under dSOC view are presented in Fig. 1 . After successful identification of the stricture in each patient, at least four single visually guided biopsies were obtained, followed by at least one BBB performed with the Spybite Max Forceps.

All endoscopists had the same step-by-step procedure form, ensuring similar biopsy techniques. Rapid on-site evaluation was not used. There was no centralization of histopathological reading; all histopathological examinations of biopsies were performed by an unblinded expert gastrointestinal pathologist at each participating center. Prophylactic measures for cholangitis and post-ERCP pancreatitis were used according to local protocols.

During the study, the biopsy protocol was adapted from bite-on-bite (double BBB) to bite-on-bite-on-bite biopsy (triple BBB) to attempt even deeper biopsies of the bile duct lesion, as initial observations did not show much promise. A set of BBB was defined as two samples for double BBB and three samples for triple BBB. Single biopsy refers to one biopsy taken from one single location, and was attempted at least four times. Examples of both biopsy techniques are shown in Video 1 .

### Data collection

Baseline data were collected and included patient demographics (age, sex), history of primary sclerosing cholangitis, IgG4-related sclerosing cholangitis, chronic pancreatitis, gallstone disease, malignancy, hepatobiliary surgery, previous placement of biliary stents, and bile duct stone removal, as well as previous diagnostic work-up regarding the IBDS. In addition, data from previous investigations, such as ERCP and EUS with tissue acquisition, computed tomography (CT), magnetic resonance imaging (MRI), magnetic resonance cholangiopancreatography (MRCP), and CA19.9 levels were collected.

During the dSOC procedure, the following variables were collected: technical success of biopsy sampling, the number of biopsies collected by each technique, and any procedural adverse events. IBDS site was divided into proximal (intrahepatic, hilar, proximal CBD, and cystic duct) and distal (distal CBD). Biopsies were evaluated and categorized by an expert pathologist into the following categories: benign, malignant, or various degrees of dysplasia (low grade or high grade), with an additional category for inconclusive results. Inconclusive results were specimens in which the pathologist was unable to interpret the tissue.

A diagnosis of malignancy could be established by dSOC-guided biopsies, other tissue-sampling methods during follow-up, surgical resection specimens, or disease progression (i. e. development of unequivocal distant metastasis) observed on cross-sectional imaging. A definitive diagnosis of a benign condition was established after a minimum follow-up period of 1 year, during which there was no confirmation of malignancy using any of the aforementioned techniques and no documented disease progression on cross-sectional imaging.

### Outcome measures

The primary outcome was the accuracy of both biopsy techniques (i. e. single biopsies and BBB) for diagnosis of malignancy in IBDS. In addition, the added diagnostic yield of performing additional BBB biopsies after single biopsies was analyzed. In the statistical analysis, specimens interpreted as low grade dysplasia and inconclusive diagnosis were considered inconclusive for malignancy; specimens interpreted as high grade dysplasia were considered positive for malignancy.

Secondary outcomes were technical success rates for obtaining tissue with both techniques and the adverse event rate of both techniques. Adverse events related to BBB, consisting of bile duct perforation and hemobilia, were graded according to the AGREE classification 16 . Other adverse events related to the ERCP or dSOC were noted separately, such as post-ERCP pancreatitis or cholangitis.

### Sample size calculation

Sample size was calculated using a two-sided McNemar’s test for paired two-sample proportion. At a significance level (α) of 0.05, 76 patients were needed to achieve the power of 0.9 (β), assuming a net benefit of 15 % of BBB compared with single biopsies.

### Data and statistical analysis

Descriptive statistical variables consisted of frequencies (%), medians, and interquartile ranges (IQRs). Sensitivity, specificity, accuracy, negative predictive value (NPV), and positive predictive value (PPV) of both biopsy techniques were calculated using 2 × 2 tables according to the final pathological diagnosis. The 95 % CIs were calculated according to the Clopper–Pearson interval. In this analysis patients who were lost to follow-up were excluded. The exact McNemar’s test was performed to compare outcomes among patients in whom both techniques were successful and final pathological diagnosis was available. A *P* value of < 0.05 was interpreted as statistically significant. All analyses were performed using R version 4.2.2. (R Foundation for Statistical Computing, Vienna, Austria).

## Results

### Baseline characteristics

A total of 89 patients with a new diagnosis of IBDS were prospectively included. The median patient age was 66 years (IQR: 55–73), and the majority of patients were male (61.8 %). The IBDS was located proximally in 76 patients (85.4 %) and distally in 13 (14.6 %). Only six patients (6.7 %) had a diagnosis of primary sclerosing cholangitis prior to dSOC. Prior work-up consisted of CT in 86 (96.6 %), MRI/MRCP in 60 (67.4 %), EUS in 21 (23.6 %) with fine-needle aspiration/biopsy (FNA/FNB) issue acquisition in 14 (66.7 %), and ERCP in 41 (46.1 %), with negative or inconclusive brushings/biopsies in all. In nine patients (10.1%), both an ERCP with tissue sampling and an EUS with FNA/FNB were performed. Overall, in 46 patients (51.7 %) prior tissue acquisition through EUS and/or ERCP was performed. All baseline characteristics are listed in Table 1 .

**Table 1:** Baseline characteristics of the included patients.

	Patients (n = 89)
Age, median (IQR), years	66 (55–73)
Female sex, n (%)	34 (38.2)
Stricture location, n (%)	
Distal CBD	13 (14.6)
Cystic duct	4 (4.5)
Proximal CBD	20 (22.5)
Hilar	38 (42.7)
Intrahepatic	14 (15.7)
CA19.9 level, median (IQR), mmol/L 1	74.4 (14.0–294.0)
**Medical history, n (%)**	
Primary sclerosing cholangitis	6 (6.7)
Chronic pancreatitis	4 (4.5)
Choledocholithiasis	10 (11.2)
Hepatobiliary surgery	10 (11.2)
Cholecystectomy	7 (70.0)
Hemi-hepatectomy	2 (20.0)
Liver transplantation	1 (10.0)
**Biliary interventions prior to dSOC, n (%)**	
Prior stent placement	37 (41.6)
Prior stone removal	7 (7.9)
**Work-up of IBDS, n (%)**	
CT	86 (96.6)
MRI/MRCP	60 (67.4)
EUS	21 (23.6)
FNA/FNB	14 (66.7)
Benign	4 (28.6)
Inconclusive	10 (71.4)
ERCP	41 (46.1)
Brush only	29 (70.7)
Benign	15 (51.7)
Inconclusive	12 (41.4)
Dysplasia	2 (6.9)
Biopsy only	1 (2.4)
Benign	1 (100)
Both brush and biopsy	11 (26.8)
Benign	3 (27.3)
Inconclusive	8 (72.7)
Prior tissue acquisition, n (%) 2	46 (51.7)
Final pathology outcome, n (%) 3	
pCCA	36 (40.5)
CBD CCA	4 (4.5)
iCCA	1 (1.1)
GBC	1 (1.1)
HCC	1 (1.1)
dCCA	6 (6.7)
Pancreatic cancer	2 (2.3)
Benign	36 (40.5)
Missing	2 (2.3)

CBD, common bile duct; CCA, cholangiocarcinoma; CT, computed tomography; dCCA, distal cholangiocarcinoma; dSOC, digital single operator cholangioscopy; ERCP, endoscopic retrograde cholangiopancreatography; EUS, endoscopic ultrasound; FNA/FNB, fine-needle aspiration/fine-needle biopsy; GBC, gallbladder cancer; HCC, hepatocellular carcinoma; IBDS, indeterminate biliary duct stricture; iCCA, intrahepatic cholangiocarcinoma; IQR, interquartile range; MRI/MRCP, magnetic resonance imaging/magnetic resonance cholangiopancreatography; pCCA, perihilar cholangiocarcinoma.

1 Measured in 69 patients.

2 Combination of ERCP and EUS.

3 Based on dSOC in 31, surgery in 18, clinical follow-up in 30, and pathological proof in 8 patients.

### Procedure characteristics

The dSOC procedure was technically successful, with visualization of the target stricture in all patients. Overall, obtaining single biopsies was technically successful in 88 /89 patients (98.9 %). In one patient, biopsies could not be obtained due to an inadequate cholangioscope position. A total of 83 patients (93.3 %) had at least four biopsies taken, and five patients (5.6%) had either two or three biopsies, which did not conform to the protocol. In 82 /88 patients (93.2 %), the pathologist concluded that sufficient tissue had been obtained for histopathological analysis.

The BBB technique was considered technically successful in 86 /89 patients (96.6 %). In three patients, biopsies could not be obtained due to inadequate cholangioscope position caused by the scope being pushed away during BBB. A median number of two sets of BBB (IQR: 2–3) were obtained. In 66 patients (76.7 %), two or more sets of BBB were obtained. In 20 patients (23.3 %) double BBBs were obtained, while in the remaining 66 patients (76.7 %) triple BBBs were obtained. Adequate tissue samples for histopathological analysis were obtained with BBB in 78 patients (87.6 %), as determined by the pathologist. No adverse events directly associated with BBB, such as hemobilia or bile duct perforation, were noted. Mild post-ERCP pancreatitis developed after dSOC in two patients, both of whom were treated conservatively (Grade II). One patient had cholangitis treated conservatively with antibiotics (Grade II), while another patient with cholangitis underwent stent exchange (Grade IIIa).

### Biopsy technique outcomes

Among the 82 patients with sufficient tissue from single biopsies available for analysis, histopathological reports revealed malignancy in 31 patients (37.8 %), high grade dysplasia in 2 patients (2.4 %), low grade dysplasia in 2 patients (2.4 %), benign disease in 38 patients (46.3 %), and inconclusive in 9 patients (11.0 %).

Among the 78 patients with sufficient tissue from BBB available for analysis, histopathological reports revealed malignancy in 29 patients (37.2 %), high grade dysplasia in 1 patient (1.3 %), low grade dysplasia in three patients (3.8 %), benign disease in 37 patients (47.4 %), and inconclusive in 8 patients (10.3 %).

**Fig. 2:**
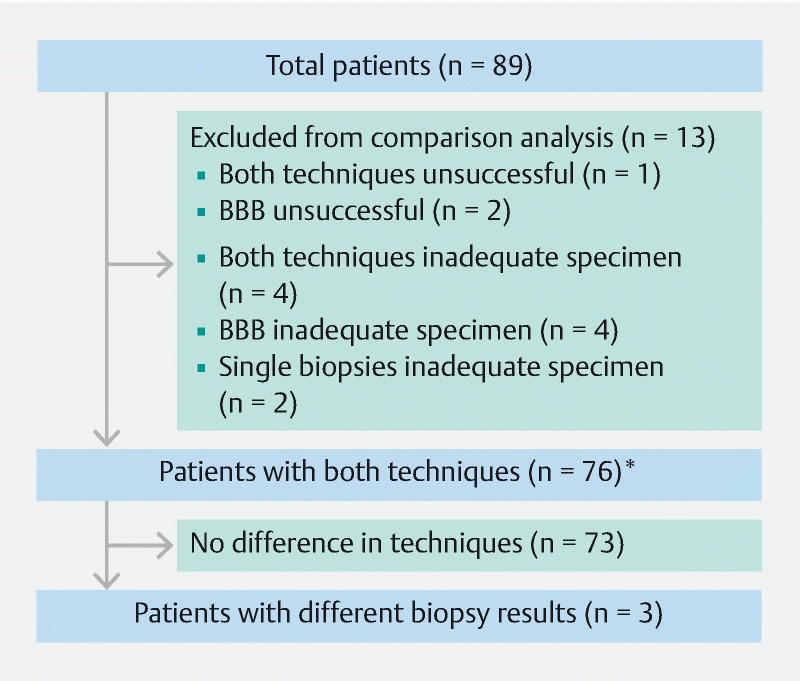
Flow chart of included patients. *All five patients with < 4 single biopsies had adequate specimens.

In 76 patients (85.4 %), both single biopsies and BBB were available and sufficient for analysis, as shown in Fig. 2 . In 73/76 patients (96.1 %) histopathological diagnosis was similar for both techniques. Three patients (3.9 %) had discordant results between the two techniques: 1) in one patient, single biopsies confirmed malignancy consistent with perihilar cholangiocarcinoma, but BBB showed benign disease; 2) in another patient, single biopsies were inconclusive, but BBB confirmed benign disease in line with clinical follow-up at > 1 year; and 3) in the remaining patient, single biopsies showed benign disease, and BBB showed low grade dysplasia, while surgical resection after 7 months identified benign sclerosing cholangitis.

BBB showed an additional yield compared with single biopsies in only 3/89 patients (3.4 %). In the abovementioned patient with inconclusive single biopsies, BBB confirmed benign disease, and in two patients in whom single biopsies were insufficient for histopathological assessment, BBB correctly confirmed benign disease.

### Final diagnosis after dSOC and follow-up

Overall, dSOC-guided biopsies confirmed malignancy in 31/89 patients (34.8 %) and high grade dysplasia in 2/89 patients (2.2 %). Among the remaining 53 patients with inconclusive, benign, or low grade dysplasia on histopathological analysis from biopsies, malignancy was confirmed after the dSOC procedure in 12/53 patients (22.6 %). This diagnosis was based on surgery in eight patients, additional ERCP-guided brushings or biopsies in two patients, additional EUS-guided tissue acquisition in three patients, and evident tumor progression on cross-sectional imaging in two patients. In the three patients with low grade dysplasia, surgical specimens confirmed benign sclerosing cholangitis in one, repeat ERCP-guided brushings showed distal cholangiocarcinoma in another, and EUS-guided tissue acquisition of the mass showed perihilar cholangiocarcinoma in the remaining patient. Regarding the two patients with high grade dysplasia in dSOC-guided biopsies, one patient had ERCP-guided biopsies with proven intrahepatic cholangiocarcinoma after 5.4 months, and the other patient had evidence of tumor progression on cross-sectional imaging after 5.1 months. In 36/56 patients (64.3 %), benign disease was confirmed through surgical resection in 9 patients and clinical follow-up in 27, with a median follow-up period of 12 months (IQR: 8–17). Two patients were lost to follow-up. 

### Diagnostic accuracy of both biopsy techniques

Overall sensitivity, specificity, PPV, NPV, and accuracy for diagnosing malignancy/high grade dysplasia in IBDS were 66.0 %, 100 %, 100 %, 63.8 %, and 78.8 %, respectively, for single biopsies, and 63.8 %, 100 %, 100 %, 63.0 %, and 77.6 %, respectively, for BBB ( Table 2 ). Accuracy was not significantly different between biopsy techniques ( *P* > 0.99). Whenever a previous ERCP with tissue acquisition was performed prior to dSOC, for both single biopsies and BBB, sensitivity, NPV, and overall accuracy were lower, as shown in Table 3 . These results were comparable to subgroup analysis with or without prior stent placement. Results regarding double or triple BBB are presented in **Table 2 s** and show a slightly higher accuracy for double BBB.

**Table 2:** Outcomes of both biopsy techniques.

	Bite-on-bite (-on-bite)	Single biopsies
Technical success, n (%)	86 (96.6)	88 (98.9)
Number of biopsies, median (IQR)	2 (2–3)	4 (4–4)
Number of biopsies, n (%)		
1	20 (23.3)	0
2	30 (34.9)	1 (1.1)
3	24 (27.9)	4 (4.5)
4	12 (14.0)	73 (83.0)
5	0	7 (8.0)
6	0	3 (3.4)
Double or triple bite, n (%)		
Bite-on-bite (double BBB)	20 (23.3)	NA
Bite-on-bite-on-bite (triple BBB)	66 (76.7)	NA
Adequate tissue, n (%)	78 (87.6)	82 (92.1)
Pathology outcome of biopsy, n (%)		
Benign	37 (47.4)	38 (46.3)
Inconclusive	8 (10.3)	9 (11.0)
Low grade dysplasia	3 (3.8)	2 (2.4)
High grade dysplasia	1 (1.3)	2 (2.4)
Malignant	29 (37.2)	31 (37.8)
Final pathology results available	76 1	80*
True positive, n (%)	30 (39.5) 2	33 (41.3) 3
False negative, n (%)	17 (22.4) 4	17 (21.3) 4
False positive, n (%)	0	0
True negative, n (%)	29 (38.2)	30 (37.5) 5
Sensitivity (95 % CI), %	63.8 (48.5–77.3)	66.0 (51.2–78.8)
Specificity (95 % CI), %	100 (88.1–100)	100 (88.4–100)
Positive predictive value (95 % CI), %	100 (88.4–100)	100 (89.4–100)
Negative predictive value (95 % CI), %	63.0 (53.9–71.4)	63.8 (54.5–72.2)
Accuracy (95 % CI), %	77.6 (66.6–86.4)	78.8 (68.2–87.1)

CCA, cholangiocarcinoma; iCCA, intrahepatic cholangiocarcinoma; IQR, interquartile range; NA, not assessable; pCCA, perihilar cholangiocarcinoma.

1 Final pathology results were missing in two patients.

2 Including one patient with high grade dysplasia and confirmed pCCA.

3 Including two patients with high grade dysplasia and confirmed CCA (1 pCCA, 1 iCCA).

4 Including six patients with inconclusive biopsies and two patients with low grade dysplasia.

5 Including one patient with no pathological proof after 370 days but moderate suspicion for pCCA.

**Table 3:** Subgroup analyses for patients with and without prior endoscopic retrograde cholangiopancreatography with tissue acquisition.

	Bite-on-bite (-on-bite)		Single biopsies	
	Prior ERCP with tissue (n = 32)	No prior ERCP (n = 44)	Prior ERCP with tissue (n = 37)	No Prior ERCP (n = 43)
True positive, n (%)	9 (28.1)	21 (47.7)	10 (27.0)	23 (53.5)
False negative, n (%)	10 (31.3)	7 (15.9)	11 (29.7)	6 (14.0)
False positive, n (%)	0	0	0	0
True negative, n (%)	13 (40.6)	16 (36.4)	16 (43.2)	14 (32.6)
Sensitivity (95 % CI), %	47.4 (24.5–71.1)	75.0 (55.1–89.3)	47.6 (25.7–70.2)	79.3 (60.3–92.0)
Specificity (95 % CI), %	100 (75.3–100)	100 (79.4–100)	100 (79.4–100)	100 (76.8–100)
PPV (95 % CI), %	100 (66.4–100)	100 (83.9–100)	100 (69.2–100)	100 (85.2–100)
NPV (95 % CI), %	56.5 (45.9–66.6)	69.6 (54.6–81.3)	59.3 (49.2–68.6)	70.0 (53.4–82.6)
Accuracy (95 % CI), %	68.8 (50.0–83.9)	84.1 (69.9–93.4)	70.3 (53.0–84.1)	86.1 (72.1–94.7)

ERCP, endoscopic retrograde cholangiopancreatography; NPV, negative predictive value; PPV, positive predictive value.

## Discussion

This study aimed to assess and ultimately improve the current biopsy techniques for diagnosing IBDS with dSOC. Although general recommendations include taking multiple single biopsies from IBDS, deeper sampling capability might offer improved diagnostic accuracy over standard single biopsies. By correct identification of the nature of the IBDS, clinical decision making and patient care are influenced. Our findings suggest that performing BBB biopsies does not increase the diagnostic accuracy in patients with IBDS. 

We found that by adding BBB to the current clinical practice of single biopsies, an additional yield was obtained in only 3.4 % of patients. In all of these patients, BBB correctly confirmed benign disease where single biopsies were inconclusive or inadequate for assessment. Direct comparison of the two techniques showed a sensitivity and specificity of 66.0 % and 100 %, respectively, for targeted single biopsies, and 63.8 % and 100 % for ≥ 1 set of BBB biopsies. Overall, these rates are in line with other reports on the yield of dSOC-guided biopsies of biliary strictures 6 17 . However, we have shown that taking one set of deeper samples by dSOC-guided BBB does not increase the yield. The rationale for taking deeper biopsies is that cholangiocarcinoma has a prominent desmoplastic and vascularized stroma. Cholangiocarcinoma has three main patterns of growth: mass-forming, periductal infiltrating, and intraductal growing 18 . In particular, periductal infiltrating cholangiocarcinoma, which may not grow into the bile duct lumen, is notoriously hard to diagnose with superficial brushes or biopsies. We discovered that BBB is more difficult to perform successfully than single biopsies, especially in patients with distal strictures, owing to scope instability and difficulty identifying the site of the first bite for BBB. Therefore, diagnostic accuracy rates found might be lower due to sampling difficulty.

It is important to note that there are many factors influencing the yield of dSOC-guided biopsies, such as the number of patients with a high pretest odds of malignancy. We performed a subanalysis in patients who underwent prior intraductal tissue acquisition through ERCP by biliary brushes and/or biopsies. For both single and BBB biopsies, the sensitivity, NPV, and accuracy were higher in patients with “naïve” IBDS ( Table 3 ). This is probably due to the selection of patients with IBDS because it may be harder to acquire diagnostic tissue from patients with previous sampling than in naïve IBDS without prior ERCP sampling. Central to this discussion is the definition of a “true” or “naïve” IBDS. While the study was not designed to evaluate dSOC as the primary method for obtaining tissue in IBDS, the high diagnostic accuracy and sensitivity of dSOC in “naïve” IBDS cases suggest its potential value. This promising data could justify further investigation of dSOC as an initial approach in index ERCP for IBDS. Future research should, however, consider the cost-effectiveness of this method. On the other hand, recent studies on optimized protocols for biliary brush cytology have shown a steep increase in sensitivity to around 78 % for perihilar and intrahepatic cholangiocarcinoma 19 .

Finally, it is important to note that historically, IBDS has been defined as biliary stricture in which prior ERCP rendered inconclusive results. Recent Asian consensus guidelines on the role of dSOC defined an IBDS to be of uncertain etiology with inconclusive/negative imaging or tissue diagnosis 1 . This definition differs critically from the recent American guidelines, which define IBDS as “one for which a diagnosis has not been established despite initial ERCP with intraductal sampling” 20 . It is important to make the distinction between undetermined and indeterminate biliary duct strictures and therefore we advocate for a uniform definition internationally, in order to decrease the heterogeneity of future studies and facilitate easier comparison 21 .

Although the Spybite Max is capable of obtaining larger tissue samples, previous studies have not indicated an increase in diagnostic accuracy 11 12 . Nonetheless, the biggest advancement in reaching definitive diagnosis will likely come from the possibility of obtaining larger tissue samples, and more studies are needed on larger biopsy forceps. Other methods of dSOC-guided tissue acquisition include snares that can remove polypoidal lesions and bile duct aspiration, aimed at circulating tumor and cell-free DNA analysis. It is likely that in the future additional methods will be developed, such as dSOC-guided brushes or dSOC-guided FNB. Meanwhile, there are many promising applications that can complement dSOC-guided biopsies, which were not taken into account in this study, such as the application of next-generation sequencing on biopsies 22 . Next-generation sequencing may provide additional information by identifying several genetic mutations associated with cholangiocarcinoma. The introduction of artificial intelligence in dSOC may guide targeted biopsies, potentially increasing the yield even further 23 24 . Another promising technique is cryobiopsy, which is designed to overcome several disadvantages of dSOC-guided biopsies, but has only been performed through the percutaneous approach 25 26 . Recently, endoscopic light-scattering spectroscopy guided by dSOC showed 97 % accuracy for detecting malignancy without tissue acquisition 27 .

A significant strength of our study is the prospective design in which patients underwent both biopsy techniques to make direct intrapatient comparison possible. We included both patients with a negative work-up after ERCP or EUS and patients who had not undergone work-up, allowing us to directly compare both groups with “true” IBDS (after an initially negative work-up) and casus pro diagnosi (without initial work-up). A structured follow-up for patients with suspected benign disease after dSOC limited the possibility of false-negative diagnosis. There are also limitations that need to be considered. First, the method of histopathological analysis, as well as the pathologists’ experience with IBDS, were not standardized. This could be improved by centralization of reading by one dedicated pathologist, but this may limit generalizability to clinical practice. Second, this study was carried out in expert treatment centers by experienced endoscopists, limiting the generalizability to lower volume centers while acknowledging that obtaining BBB biopsies is technically challenging. Third, several characteristics of the IBDS itself, such as length of the stricture and visual characteristics during dSOC, were not collected systematically. Fourth, we included high grade dysplasia as a positive outcome together with malignancy, because high grade dysplasia is treated as being malignant in the participating treatment centers. Finally, dSOC is an expensive procedure and there is still limited information pertaining to its cost-effectiveness and its environmental footprint. Finally, we did not differentiate between the various types of cholangiocarcinoma. It is conceivable that there is a differential yield between exophytic and non-exophytic types.

In conclusion, obtaining a set of biopsies through BBB did not produce statistically significant differences in diagnostic yield compared with the current strategy of multiple single biopsies. We cannot conclusively refute the hypothesis of equivalent accuracy between the two strategies. An interesting approach to assessing the value of BBB would be to collect all consecutive “bites” of the BBB in separate containers to determine the true added value and possibly the optimal number of consecutive biopsies at one site in IBDS. Therefore, further studies with larger sample sizes are needed, assessing the successive yield of BBB per set, for multiple sets, and stratified by cholangiocarcinoma type, including the addition of next-generation sequencing and a larger biopsy forceps.

## Supplementary Material

Supplementary material Zusatzmaterial
